# Structural Organization of DNA in Chlorella Viruses

**DOI:** 10.1371/journal.pone.0030133

**Published:** 2012-02-16

**Authors:** Timo Wulfmeyer, Christian Polzer, Gregor Hiepler, Kay Hamacher, Robert Shoeman, David D. Dunigan, James L. Van Etten, Marco Lolicato, Anna Moroni, Gerhard Thiel, Tobias Meckel

**Affiliations:** 1 Plant Membrane Biophysics, Technische Universität Darmstadt, Darmstadt, Germany; 2 Computational Biology Group, Technische Universität Darmstadt, Darmstadt, Germany; 3 Department of Biomolecular Mechanisms, Max Planck Institute for Medical Research, Heidelberg, Germany; 4 Department of Plant Pathology and Nebraska Center for Virology, University of Nebraska, Lincoln, Nebraska, United States of America; 5 Department of Biology and CNR IBF-Mi, Università degli Studi di Milano, Milano, Italy; University of Kansas Medical Center, United States of America

## Abstract

Chlorella viruses have icosahedral capsids with an internal membrane enclosing their large dsDNA genomes and associated proteins. Their genomes are packaged in the particles with a predicted DNA density of ca. 0.2 bp nm^−3^. Occasionally infection of an algal cell by an individual particle fails and the viral DNA is dynamically ejected from the capsid. This shows that the release of the DNA generates a force, which can aid in the transfer of the genome into the host in a successful infection. Imaging of ejected viral DNA indicates that it is intimately associated with proteins in a periodic fashion. The bulk of the protein particles detected by atomic force microscopy have a size of ∼60 kDa and two proteins (A278L and A282L) of about this size are among 6 basic putative DNA binding proteins found in a proteomic analysis of DNA binding proteins packaged in the virion. A combination of fluorescence images of ejected DNA and a bioinformatics analysis of the DNA reveal periodic patterns in the viral DNA. The periodic distribution of GC rich regions in the genome provides potential binding sites for basic proteins. This DNA/protein aggregation could be responsible for the periodic concentration of fluorescently labeled DNA observed in ejected viral DNA. Collectively the data indicate that the large chlorella viruses have a DNA packaging strategy that differs from bacteriophages; it involves proteins and share similarities to that of chromatin structure in eukaryotes.

## Introduction

Chloroviruses in the family *Phycodnaviridae* have a long evolutionary history possibly dating back to the time when eukaryotes arose from prokaryotes [1]–[3]. They are predicted to have a common ancestor with the poxviruses (e.g., vaccinia virus), asfarvirues, iridoviruses, ascoviruses and mimiviruses [1], [4], [5]. Collectively, these viruses are referred to as nucleocytoplasmic large DNA viruses (NCLDVs).

PBCV-1 virions, the prototype chlorovirus, are large icosahedral particles (190 nm in external diameter) that have an internal lipid bilayered membrane [6]. However, the particles have more surface features than was originally thought. One of the PBCV-1 vertices has a 560 Å long spike structure; 340 Å protrudes from the surface of the virus. The part of the spike structure that is outside the capsid has an external diameter of 35 Å at the tip expanding to 70 Å at the base [7], [8]. The spike structure widens to 160 Å inside the capsid and forms a closed cavity inside a large pocket between the capsid and the internal membrane enclosing the virus DNA. Therefore, the internal virus membrane departs from icosahedral symmetry adjacent to the unique vertex. Consequently, the virus DNA located inside the envelope is packaged non-uniformly in the virion. In addition to the spike, external fibers extend from some virus capsomers.

PBCV-1 infection resembles infection by tailed bacteriophages because its genome must cross the cell wall (and membrane) of its host *C. variabilis* to initiate infection. The PBCV-1 spike first contacts the host cell wall [8] and the fibers aid in holding the virus to the wall. The spike is too thin to deliver DNA and so it probably serves to puncture the wall and is then jettisoned. Following expansion of the hole in the host wall by a virus-packaged enzyme(s), the viral internal membrane presumably fuses with the host membrane, facilitating entry of the PBCV-1 DNA and virion-associated proteins into the cell, leaving an empty capsid attached to the surface [9]. This fusion process triggers rapid depolarization of the host membrane [10], possibly by a virus encoded K^+^ channel (named Kcv) predicted to be located in the internal membrane of the virus, followed by rapid release of K^+^ from the cell [11] and altered secondary active transport of solutes [12]. The rapid loss of K^+^ and associated water fluxes from the host reduce its turgor pressure, which may aid ejection of viral DNA and virion-associated proteins into the host [13].

A property that all NCLDVs including the chloroviruses share with dsDNA bacteriophages and other DNA viruses is that they package a dsDNA genome into a geometrically confined capsid. An example of DNA packaging is the 48.5 kb genome of bacteriophage λ. Its extended linear form of 16.5 µm [14] is compressed into a capsid with an inside radius of 27.5 nm [15], creating a DNA density inside the particle of 0.6 bp nm^−3^. This value approaches the maximal theoretical density for DNA packaging and the DNA is almost at crystalline density inside the phage head [16]. A lower DNA packaging density occurs in the two NCLDVs, vaccinia virus and mimivirus. Both viruses package their large DNA genomes with a density of ∼0.05 bp nm^−3^ [17], [18]. Our estimates of DNA packaging density place the chlorovirus PBCV-1 between phage λ and vaccinia virus and mimivirus. The ∼330 kb genome of virus PBCV-1 is compressed into a capsid with an inner radius of about 72 nm providing a DNA density of ∼0.2 bp nm^−3^.

DNA packaging density has implications for virus infection. Experiments and theoretical calculations indicate that the high DNA packaging density in phages generates enormous internal pressure in the particles ranging up to 50 bars [19]. This pressure serves as a driving force for the rapid ejection of DNA from the virus particle. For example, phage λ expels its DNA with an initial velocity of 60 kbp/sec, which then decreases as the residual amount of DNA in the particle decreases [20]. As a result, the entire DNA can be propelled from the capsid in ∼1.5 sec under optimal conditions [14]. In another example, phage T5 DNA is expelled in a stepwise fashion at a rate reaching 75 kpb/sec [21]. This pressure driven DNA ejection provides at least part of the energy required for transfer of the DNAs into their hosts [22].

PBCV-1 is unique among the NCLDVs in that it uncoats its DNA at the cell surface and leaves an empty capsid on the outside of the cell wall, similar to many tailed bacteriophages. Consequently, PBCV-1 may use similar mechanical forces to eject its genome into its host cell as phages [13]. In contrast, most NCLDVs are not faced with a cell wall and they initiate infection by either an endocytotic or an envelope fusion mechanism with the host plasma membrane; they then uncoat inside the cell. Consequently, most NCLDVs do not require a high DNA density to initiate infection. In fact, when DNA is released from the vaccinia capsid it does not burst out but rather pours out like a thick fluid [17], suggesting that forced ejection of vaccinia DNA is not important for its infection.

Phage DNA packaging depends primarily on two parameters, the function of motor proteins and cations. Dense packaging of DNA requires that 90% of its charge is neutralized [23]. Evidence for charge neutralization of densely stored DNA in phages existed more than 50 years ago. While phages typically use cations to neutralize their DNA some phages use polyamines, such as putrescine and spermidine in addition to cations [24]. There is no evidence indicating basic proteins contribute much to neutralizing phage dsDNA genomes [25], [26]. Indeed the dense DNA-packaging in many phages leaves little room for DNA binding proteins [27]. As mentioned above, DNA packaging in NCLDVs faces similar challenges to those in bacteriophages. Indeed many dsDNA viruses use proteins for DNA packaging. *Polyomaviridae* [28] and *Papillomaviridae* [29] for example can functionally co-opt host histone proteins. Other dsDNA viruses (*Adenoviridae*, *Asfarviridae*, *Baculoviridae*) express small arginine rich protamine-like proteins with putative DNA condensation functions [30], [31].

Currently little information is available on the mode of DNA packaging in the large chloroviruses. At least in the case of PBCV-1, and presumably the other chloroviruses, DNA neutralization may also employ proteins because PBCV-1 virions contain 148 different viral-encoded proteins (Dunigan et al., manuscript in preparation), some of which have been described as DNA-binding proteins [32]. This large number suggests that DNA binding proteins play a role in the organization and packaging of chlorovirus DNA genomes. Here we report a procedure for releasing PBCV-1 DNA from the virus particle and analyze its structural properties.

## Results and Discussion

### Ejection of PBCV-1 DNA from capsids

Phage DNA release is often triggered by an interaction between phage tails and host receptor. This interaction causes the DNA to rapidly expand and the DNA catapults out of the capsid [14]. The host receptor for virus PBCV-1 is unknown, although circumstantial evidence suggests it is carbohydrate [33]. Still DNA release from PBCV-1 can be achieved by infecting *C. variabilis* cells with a high m.o.i. (e.g., 100). Under these conditions it occurs as if the DNA is released from the virus particle but not able to enter the host; as a consequence the particle is dynamically catapulted away from the host cell leaving an unraveled, quasi-linear DNA polymer tethered to the cell. In some images it is possible to see the capsid at the end of the DNA thread projected away from the host (data not shown). The reason for the release of DNA into the medium is not known. However we know from other studies that usually only one virus infects the host cell, while the remaining viruses are excluded [34]. The fact that DNA release into the medium is only apparent at very high m.o.i. suggests that most viruses under these circumstances are not able to eject their DNA into the host because of an yet unknown exclusion mechanism.

**Figure 1:**
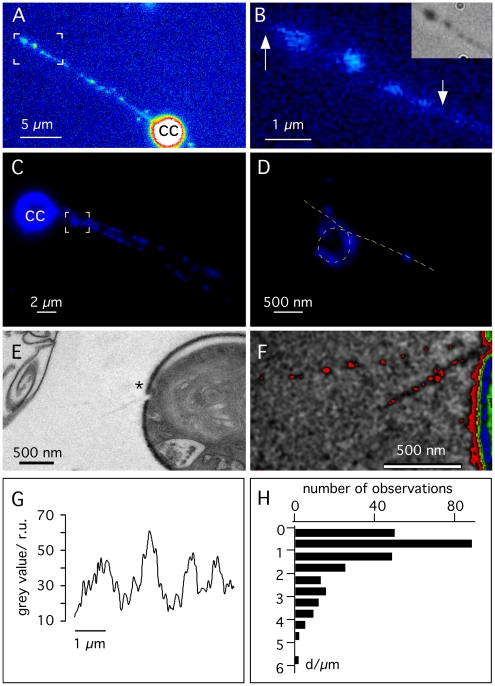
Ejection of viral DNA. **A:** Fluorescence images of *C. variabilis* with ejected DNA molecules. The incubation medium contained *C. variabilis* cells and virus PBCV-1 at an m.o.i. of ∼100 plus the fluorescent DNA stain DAPI. The image shows a chlorella cell (cc) and the viral DNA molecule, which is propelled away from the alga cell. **B:** Magnification of the area indicated by the box in **A. Inset:** same area as in **B** with conventional light microscopy and phase contrast. **C:** same as in **A** but with two DNA bands projecting away from a chlorella cell (cc). **D:** Magnification of area indicated by box in **C** with loop like DNA structure. **E:** Electron micrograph of viral DNA projecting away from host cell wall. The cell wall of the alga exhibits the typical hole (*), which the viruses digest for infection. From this hole two linear structures project towards the left side. The part marked in **E** is magnified in **F** and presented in artificial colors in order to highlight the linear structures projecting away from the cell wall hole. **G:** fluorescence intensity profile along DNA molecule between arrows in **B**. **H:** Histogram of distances between individual fluorescence maxima as in **E** from 30 ejected DNA molecules.

This release of DNA into the medium must be fast because it is possible to detect isolated DNA molecules already within 5 min post infection. Figure 1A shows a fluorescent image with a host cell and the unfolded DNA polymer from a virus particle. The DNA dye DAPI produces bright staining of the nucleus of the chlorella cell in the lower right part of the figure; in the upper left part of the figure the unraveled DNA from a virus capsid protrudes as a nearly linear structure from the cell. In the case of phages it has been argued that the release of the genome can be explained on the basis of Brownian motion [35]. Such a process however cannot account for the observed DNA ejection from virus PBCV1. Brownian movement would not generate the sort of straight lines and would also be much slower [22]. Hence the present data stress the importance of an osmotic pressure in the dense environment of the capsid, which creates a driving force for DNA ejection. In this sense PBCV-1 DNA ejection into the medium resembles that of many phage genomes [14], [21]. The forceful ejection of DNA from the PBCV-1 particle is consistent with the concept that PBCV-1 depends, at least to some extent, on these mechanical forces to eject its DNA against the turgor pressure of the chlorella host cell [13].

Frequently we observed two DNA strands under the same conditions, which projected away from the host at a common point of origin (Fig. 1C). This phenomenon was not only observed once but in ∼20% of the ejected DNA molecules. Since the surface of a Chlorella cell is ca. 500 times larger than that of a virus it is statistically rather unlikely that two virus particles independently infect at an m.o.i of 100 a host cell so frequently in the same spot. Hence it is more likely that the two DNA polymers were not from separate viruses but from a single virus. This interpretation is supported by the electron microscopic images depicted in Figs. 1E, F. These images show a *C. variabilis* cell with the typical hole in the wall, which a virus digests in the course of infection. From this location two linear structures project away from the host in an angular fashion. The projecting structures are most likely unfolded viral DNA because the half width of their cross section is <10 nm. The combination of electron microscopic and fluorescent images suggests that PBCV-1 DNA might not in all cases enter its host initially by either of its termini. This scenario would suggest that packaging of the DNA in the virion differs from ejection because it is unlikely that DNA packaging begins in the middle of the genome.

### Virus PBCV-1 DNA has structure

Close scrutiny of the fluorescently labeled ejected PBCV-1 DNA suggests it is structured. The images indicate: i) a non-uniform distribution of DNA and ii) loops in the DNA polymer. Images of ejected DNA at higher magnifications indicate that the fluorescence associated with the DNA exhibits distinct maxima. Fig. 1B shows part of an enlarged fluorescent DNA band from Fig. 1A. The fluorescence signal alternates between high and low fluorescence intensity along an imaginary line (Fig. 1B). The locations of the intensity maxima coincide with structures, which occasionally can be seen with phase contrast in a light microscope (inset Fig. 1B). This observation implies that the non-uniform fluorescence of DAPI staining is not caused by a preference of the dye to interact with A-T rich regions in the DNA but instead the intensity maxima are due to local concentrations of DNA.

In regions where the fluorescence signal was well resolved the intensity maxima were quasi periodic (Fig. 1G). In this example, the maxima were ca. 1.2 µm apart. The distances between fluorescence peaks measured in 30 individual ejected DNA molecules are summarized in Fig. 1H. The histogram shows a broad distribution of gap sizes. The distribution has a maximum below 1 µm and possibly a second one below 3 µm (Fig. 1H).

The structured pattern seen with PBCV-1 DNA does not occur in fully ejected DNA from phages λ or T5; the phage DNAs fluoresce homogeneously along the axis of the extended polymer [14], [21]. Collectively, these results suggest that the distribution of fluorescent maxima is an inherent property of PBCV-1 DNA structure. Hence PBCV-1 DNA molecules in the capsid are apparently organized differently than those in the two phages. The PBCV-1 DNA is most likely concentrated in a periodic fashion in submicroscopic coils.

In addition to these periodic domains of concentrated DNA, we occasionally detected another higher order organization in the PBCV-1 DNA strands (Figs. 1C, D); in this case, the polymer has a large loop on which several concentrated domains are clustered. The diameter of this loop is ∼600 nm; similar loops with a mean diameter of 550±100 were detected in four other images.

Collectively, the data suggest that PBCV-1 DNA is stored inside the capsid in an ordered fashion. This structure includes a periodic formation of folds and on a larger scale a formation of loop structures. These loop structures probably open up during the ejection and are hence observed only in rare cases.

### Examination of PBCV-1 DNA by AFM

**Figure 2:**
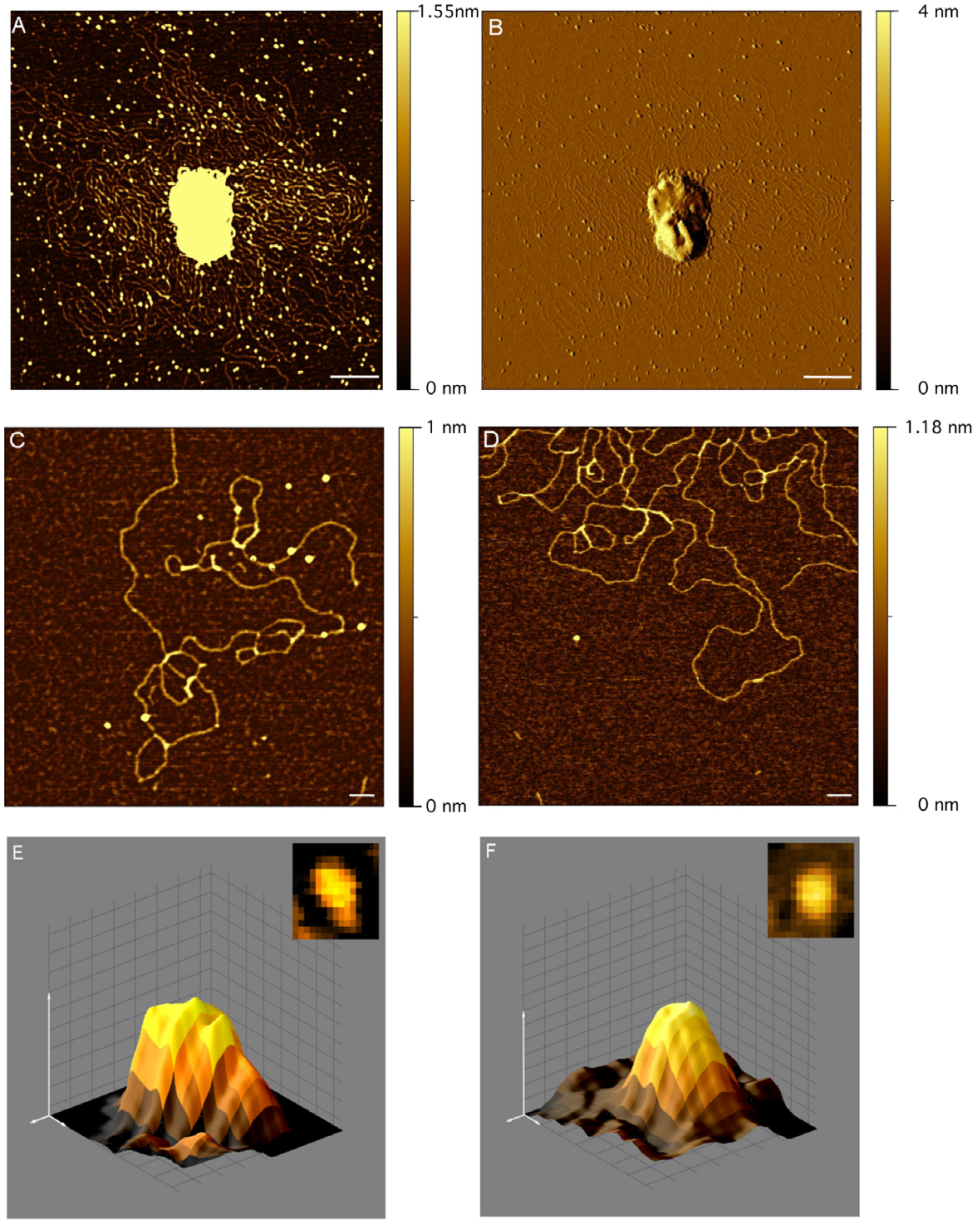
AFM images of viral DNA and associated proteins, which were isolated by osmotic shock. Scan of single PBCV-1 particles after osmotic shock in a height image **A** and in amplitude image **B**. The images reveal emerging DNA and protein particles from the disrupted virus. Magnification of DNA from disrupted virus with protein particles **C**. Proteins are absent after the sample was treated with proteinase K **D**. 3 dimensional image of individual BSA protein **E** and of individual purified 70 kDa PBCV-1 protein A278L. The latter is a putative DNA-binding protein coded by virus PBCV-1. Scale bars 100 nm in A–D and 2 nm in E and F.

To obtain more information on PBCV-1 DNA structure we viewed the isolated DNA by AFM. For these experiments PBCV-1 particles were osmotically shocked and subsequently transferred with the emerging content onto fresh mica for imaging. This procedure separates proteins that are tightly bound to DNA from those that are free. Figure 2A shows a typical ruptured particle and its associated content. Higher magnification shows the emerging DNA together with numerous particles (Fig. 2B, C). These particles are not observed with pure plasmid DNA (results not shown); hence they are specific to the virus preparation. The particles contain proteins because the particles disappear in the AFM images after treating the preparation for 1 min at 37°C with protease K (1 mg/ml) (Fig. 2D). This interpretation is supported by the fact that PBCV-1 virions contain many basic proteins (see below).

Association of DNA with proteins was reported in a previous AFM study on ruptured PBCV-1 particles [36]. The association of proteins with the DNA is not random. For example, in the Fig. 2C image DNA occupies ∼5% of the total image area but 50% of the proteins are directly associated with the DNA molecule. A similar bias of DNA and proteins occurred in other images analyzed in the same manner. The intimate association between DNA and proteins is also supported by force measurements. When a protein particle was pulled from the surface with the cantilever, the force was about 100 times higher for particles associated with DNA than for free particles.

A comparison between images of PBCV-1 DNA shows that proteins associated with DNA in AFM images produce a different periodic pattern than the fluorescence images. The reason for this difference in DNA/protein association is probably related to the isolation method. The image in Fig. 2B suggests that the osmotic shock method results in a more violent and unorganized release of DNA from the virus capsid than does the ejection method. Consequently, the osmotic forces probably disrupt the more delicate DNA structure. This is consistent with the observation that the typical periodic structure of DAPI-labeled DNA, as in Fig. 1, disappears when the DNA is released by an osmotic shock as in Fig. 2 (data not shown).

To estimate the size of the proteins associated with PBCV-1 DNA, we imaged the volume of a large number of DNA associated protein particles (Fig. 2C, F); this is possible because molecular volumes correlate with the molecular mass of proteins [37]. Measurements were first made on the 66 kDa bovine serum albumin (BSA) protein (Fig. 2E) and a recombinant expressed 69 kDa putative PBCV-1 DNA binding protein (CDS A278L) to calibrate the system (Fig. 2G). The estimated volumes of the BSA and A278L proteins were ∼57.6±0.13 nm^3^ (435 measurements) and 66.4±0.09 nm^3^ (652 measurements), respectively. Measurements of 711 randomly chosen protein particles associated with the disrupted virus produced a mean volume of 60.3±0.09 nm^3^. These experiments suggest that the PBCV-1 DNA particles are associated with a protein(s) in the range of ca. 60 kDa. The resolution of the images does not allow one to distinguish between monomers of a 60 kDa protein or multimers of smaller proteins.

To estimate the ratio of proteins that associate with DNA from the virus particles, we measured the length of the DNA molecule versus the number of total proteins in 5 images (e.g., Fig. 2B). For this analysis we considered all spherical particles that exceeded background noise by a factor >2 irrespective of whether they were free or associated with DNA. This analysis produced a number of 0.018±0.005 proteins per nm of DNA, which translates into one protein per ∼55 nm of DNA. This number implies that the entire DNA from a PBCV-1 virion, which is ∼100 µm long in its extended form, should be associated with ≥2,000 proteins. This number is an absolute minimum estimate because some proteins are probably lost during the preparation of the DNA and small proteins are masked by background noise.

### PBCV-1 DNA binding proteins

**Figure 3:**
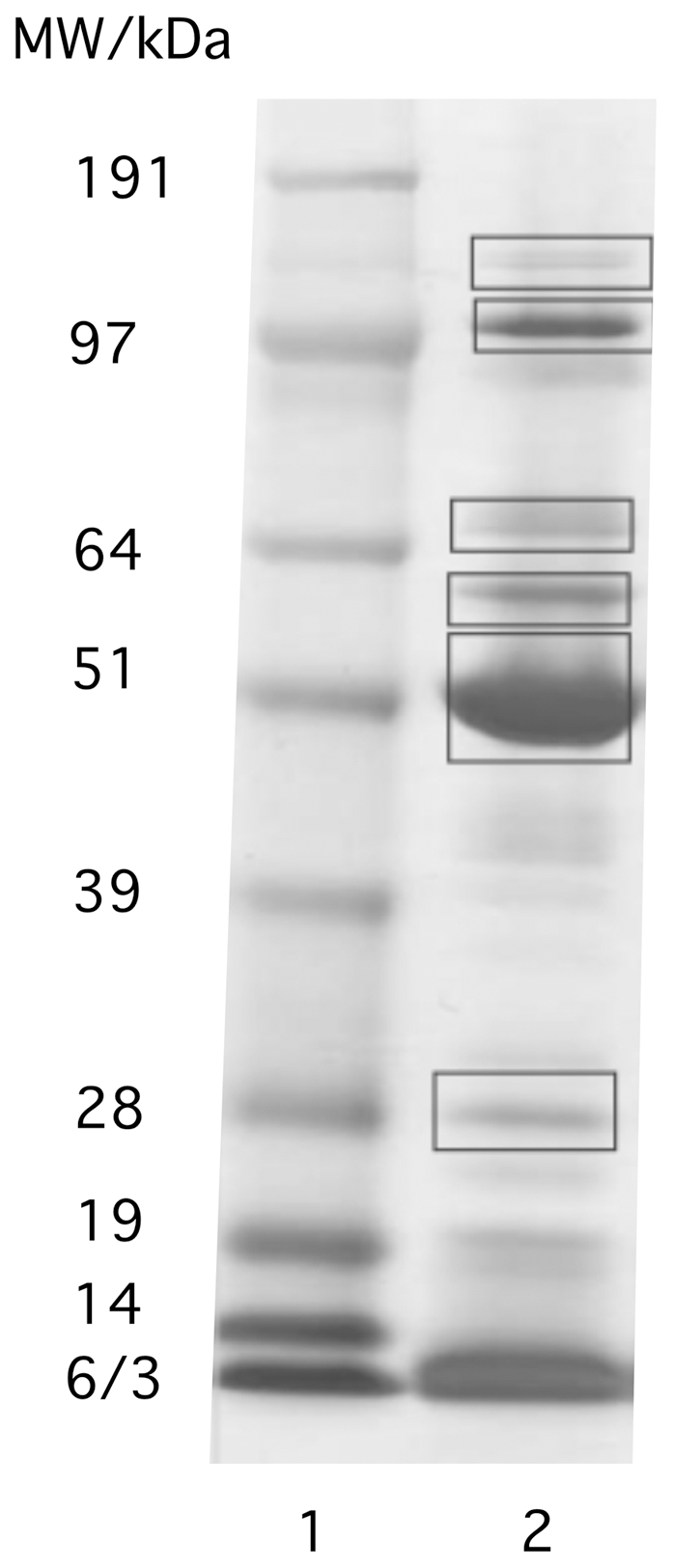
SDS-PAGE pattern of proteins associated with PBCV-1 DNA. DNA was released from capsids by osmotic shock and separated from soluble proteins by centrifugation. The DNA-containing pellet was treated with DNase to release DNA-bound proteins. The framed bands were excised and used for MALDI TOF analysis. Lane 1: weight marker, lane 2: proteins obtained after DNAse treatment.

Taken together the results from the two preceding sets of experiments suggest that PBCV-1 DNA is packaged differently than phages and it is probably associated with proteins. Even though PBCV-1 does not contain canonical histones an association of DNA with proteins is supported by analysis of the proteins packaged in a related chlorovirus CVK2 [32]. This study reported that the virions contain 7 DNA binding proteins of which 3, with estimated molecular weights of 63, 42 and 25 kDa, had high affinity for the viral DNA. To extend this previous study to PBCV-1, viral DNA was released by osmotic shock and the DNA with associated proteins was separated from soluble proteins by centrifugation according to [38]. This procedure led to about a 30-fold concentration of DNA in the pellet fraction. The pellet with the DNA and DNA-associated proteins was re-suspended in buffer containing DNAse, incubated for 1 h, and the samples were electrophoresed on SDS-polyacrylamide gels. Several prominent protein bands were detected (Fig. 3). The 6 most prominent protein bands were excised from the gel and subjected to PMF using trypsin. The peptides were analyzed by MALDI-TOF and matches to PBCV-1 proteins were identified with Mascot Server software. All database searches were also performed against the Mascot Server automatic decoy database. The latter generates a random set of sequences with the same amino acid composition as the authentic database entries. The searches with the decoy database were negative (no significant matches) except for the search with the peptide mass list that identified CDS A523R. In this search, A523R (19 kDa) was the single match with a protein score of 122 (cutoff of 43) (expect value of 6.7 e^−10^) and one match in the decoy database occurred with a protein score of 48 (cutoff of 43). From the spectra, 9 viral proteins were identified with a significance of p<0.05 (Table 1). Among these 9 proteins are 2 abundant PBCV-1 capsid proteins (CDSs A430L and A140/145R), which almost always occur as contaminates when fractionating PBCV-1 proteins; they were eliminated as important DNA-binding candidates. Of the remaining 7 proteins, 6 have high isoelectric points, which are well suited for binding and neutralizing DNA. A bioinformatics prediction of DNA binding sites in these proteins revealed several putative interactive sites between the proteins and DNA (Table 1). These experimental results are consistent with the finding that the PBCV-1 virion contains many basic proteins that are suitable for binding/neutralizing the viral DNA and which are associated with the DNA even after isolation. Of these 6 possible PBCV-1 DNA binding proteins, two have molecular weights of ∼70 kDa [A278L (69 kDa) and A282L (63 kDa)]. In addition, a dimer of A284L (60 kDa) and a trimer or tetramer of A523R (57 or 78 kDa) could produce a protein of ∼70 kDa. It is interesting that both A278L and A282L have protein kianse activity (Paola Valbuzzi unpublished results). Also one of the virus DNA binding proteins identified by [32] had protein kinase activity. This putative dual function of the DNA binding proteins is interesting in the context of small proteins from *Baculoviridae*, which bind and dissociate from the viral DNA depending on their phosphorylation state [39]. The inherent kinase activity of the putative DNA binding proteins in the chloroviruses could have a similar regulatory importance for DNA condensation.

**Table 1:** Identification of DNA binding proteins and most abundant proteins in PBCV-1 virion from MALDI-TOF PMF analysis.

ORFs	EmPAI Exponentially-modified protein abundance index	Copy number per virion	MW/kDa	%DNA binding	IP/pH	annotation
**A548L**	0.06	7	57.4	19	9.5	SWI/SNF-related matrix-associated actin-dependent regulator of chromatin subfamily A member 5
**A189/192R**	0.5	55	143.6	39	11.4	Chromosome remodelling complex
A561L	1.44	159	71.0	n.d.	9.9	unknown
**A363R**	2.01	222	128.4	36	10.9	Similar to D6/D11-like helicase(Marseillevirus) (6e-06)
**A284L**	2.41	266	30.8	20	9.2	Choloylglycine hydrolase
A035L	2.48	273	65.6	n.d.	8.9	unknown
A565R	2.49	275	73.2	n.d.	7.3	unknown
**A140/145R**	3	331	120.9	35	11.0	Located at the unique vertex of the virus particle
**A278L**	3.6	397	69.2	36	10.8	Protein kinase domain/PBCV-1 specific basic adaptor domain
**A282L**	4.83	533	63.4	35	10.8	Protein kinase domain/PBCV-1 specific basic adaptor domain
**A430L**	13.06	1440	48.2	48	7.5	Large eukaryotic DNA virus major capsid protein
A437L	19.75	2178	10.9	n.d.	11.0	Non-histone chromosomal protein MC1
**A523R**	24.79	2733	19.1	18	9.6	unknown

The table lists (in bold) the PBCV-1 CDSs from which peptides were detected in MALDI-TOF spectra. The peptides correspond to DNA associated proteins described in Fig. 3. Only database entries with a protein score with a significance <0.05 are presented. The identity of the A278L protein was confirmed by the MS/MS sequencing of 3 peptides. Also included are the copy numbers per virion of the most abundant proteins in PBCV-1 virion. The copy numbers were estimated using the exponentially modified protein abundance index (emPAI) algorithm [40]. Based on the knowledge that the major capsid protein (A430L) is present in 1440 copies per virion, we calculated the abundances of the major proteins in the virion. For these proteins, parameters such as the molecular weight (MW), the isoelectric point (IP) and the functional annotation from the Greengene database (http://greengene.uml.edu/database/php/get_orf.php?genome_name=PBCV1) are provided. Putative DNA binding sites were further identified by the BindN algorithm (http://bioinfo.ggc.org/bindn). The data are presented as percentage of putative DNA binding sites relative to total protein (% DNA binding).

In a separate experiment, we determined the proteome of the entire virus particle (Dunigan et al., manuscript in preparation) and estimated the most abundant proteins in the particle using the exponentially modified protein abundance index (emPAI) algorithm [40]. Based on the assumption that the major capsid protein (A430L) is present in 1440 copies per virion, we estimated the abundances of the major proteins in the virion (Table 1). The data show that some of the proteins are present with copy numbers in the range of several hundred to 2,000. Interesting to note is that some of the most abundant proteins again are very basic; some of these abundant proteins were also detected in association with the DNA, including A278L, A282L, A284L and A523R (Table 1). In addition, one host encoded histone-like protein was detected in the virion that appears to be present in low amounts.

### Identification of potential protein binding domains in PBCV-1 DNA

**Figure 4:**
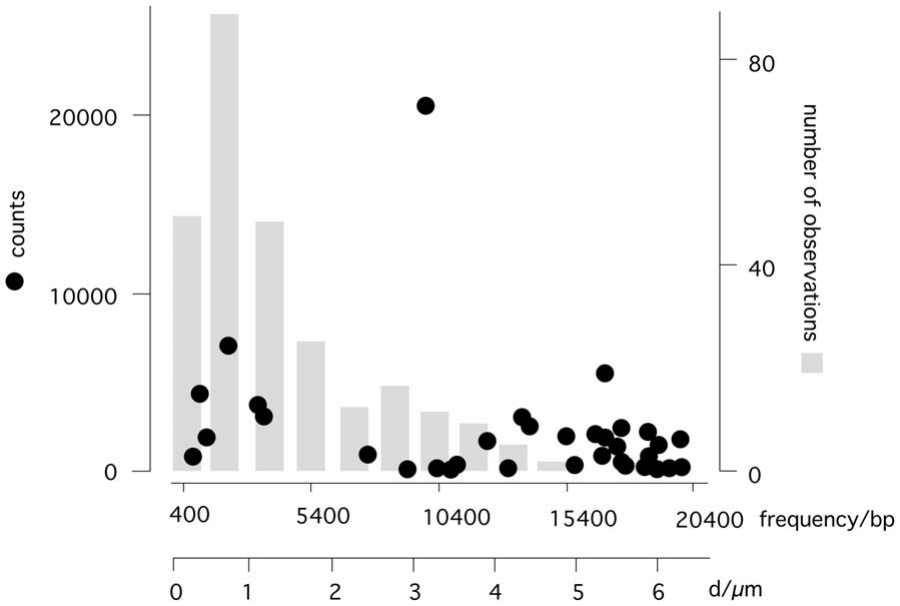
Histogram of the leading inverse Fourier-frequency (in bp) for all sequence motifs of length eight (round symbols). The most pronounced peaks occur at 9,935 bp, 2,138 bp, and 17,020 bp. For comparison we also illustrate the distribution (grey bars) of distances between individual fluorescence maxima from Fig. 1H. We assume that 1 kb DNA is in the extended form 0.323 µm long.

The periodic pattern of isolated DNA bands reported in Fig. 1 prompted us to develop a Fast-Fourier-Transformation (FFT) protocol (see Materials and Methods and Bioinformatics Analysis S1) to identify potential periodicities of binding motifs in the PBCV-1 sequence. We chose the Hamming distance with respect to a given motif and averaged over all motifs of a given length. This procedure revealed Fourier components spanning lengths of 9,935 bp, 2,138 bp, and 17,020 bp that were the three major contributors to the Fourier series expansion of the Hamming distance data set (Fig. 4). These periodic bp patterns translate into distances of 3.2 µm, 0.7 µm and 5.4 µm. Hence, the PBCV-1 genome exhibits a pattern, which roughly resembles the distribution of fluorescence maxima along the isolated DNA polymer. The most frequent spanning lengths of 2,138 bp and 9,935 bp coincides with the two maxima in the distribution of the gaps between fluorescent maxima below 1 and 3 µm (Fig. 4). The fact that the measured amplitudes at corresponding frequencies between fluorescent maxima does not correlate with the calculated frequency of spanning length distances between putative binding motives in the genome may suggest that the different motives have different binding affinities or binding specificity for DNA binding proteins (Fig. 4).

According to the distribution of Fourier-amplitudes (Bioinformatics Analysis S1), the most pronounced sequence motifs are significantly distant from a random, average sequence motif. We note that the results were similar using six and eight bp-size motifs, thus robust under motif length change.

This protocol has an additional advantage because it can conceptually reveal periodicities of much larger motifs; e.g., consider the simple case of two motifs separated by some variable genomic region. Separately these two motifs would each exhibit the same periodicity. If we analyze the data averaged over all motifs, however, we also reveal the “synchronization” of the motifs along the chain and therefore the correlated periodicity.

To verify our results and to demonstrate the robustness of our protocol we repeated the analysis using a strict criterion: an exact match between the motif without wildcard characters and the genomic fragment. As mentioned in the Materials and Methods section, this method is not as sensitive with smaller motifs; therefore, we restricted our motif length to six bps. We then repeated the FFT on this data set using the strict criterion. The experiment produced conclusive results with inverse frequencies of 9,571 bp, 17,300 bp and 16,801 bp; in addition we also obtained a signal at 6,835 bp. Details on the procedure and on the most important motifs for the Hamming distance FFT are listed in Bioinformatics Analysis S1. Interestingly, all possible CG-combinations contributed to a large extent, while those with A or T nucleotides were less significant, that is periodic. The data also show that there is a large ratio of the FFT-coefficients, thus indicating the significant different pattern of periodicity of CG- vs. “some-AT”-regions.

### Conclusions

The present results suggest that chlorovirus PBCV-1, like their eukaryotic hosts, neutralize their DNA with DNA binding proteins. The data are consistent with a model in which the viral genome has an inherent pattern with periodically spaced GC rich regions, which provide interactive sites for DNA binding proteins. DNA is presumably wound around the respective basic proteins for neutralization and packaging. The interaction results in the isolated DNA containing periodic thickenings; a higher order of organization may also involve small loops, which contribute to packaging DNA and/or gene regulation. The robust aggregation of the DNA with proteins also favors a stable structure for the virus DNA when it is ejected into the cytosol of the host, where the cation concentration is reduced [11]. The current results do not distinguish if this organization of DNA is contributing to the neutralization of the entire DNA polymer or if it is just a component of its meta-organization with the goal to achieve a crystalline-like or ordered structure inside the virion. The data however indicate that the chlorella viruses and possibly other large DNA viruses have developed a DNA packaging strategy, which involves proteins and hence shares similarities to that of chromatin in eukaryotes.

## Materials and Methods

### Materials

*Chlorella* NC64A (recently named *Chlorella variabilis* [41]) and virus PBCV-1 were grown and isolated as described previously [42]. Viral DNA was isolated from particles by two procedures: i) hyper-infection and ii) by osmotic shock. In the first procedure, isolated PBCV-1 particles were incubated for 10 min in standard modified Basic Bolds medium (MBBM) [42] with 30 µM 4′,6-Diamidino-2-phenylindol (DAPI) to label its DNA. *C. variabilis* cells were inoculated in MBBM containing 30 µM DAPI with the fluorescently labelled PBCV-1 at a multiplicity of infection (m.o.i) of ∼100 [42]. This process leads to the dynamic release of DNA from a few virus capsids. For DNA release via osmotic shock, a PBCV-1 suspension (8×10^10^ PFU/ml) was incubated for 1 h in 0.5 M KCl solution and then rapidly transferred to 60 mM KCl. For microscopic imaging experiments, the DNA was then labelled by adding 30 µM DAPI to this solution. For other experiments, we transferred the particles to fresh mica for atomic force microscopy (AFM) imaging or kept the diluted solution for 1 h before separating the soluble proteins from the released DNA according to [38]. This procedure resulted in the viral DNA being concentrated in the pellet by a factor >30. The pellet containing DNA and associated proteins was re-suspended in 10 µl of distilled water.

### Fluorescence microscopy

PBCV-1 particles and ejected fluorescently-labelled DNA were imaged on a Zeiss Axioskop 40™epifluorescence microscope. Samples were excited at 358 nm and fluorescence detected through a 461 nm filter. The images were recorded with a sensitive electron multiplying charged coupled device and a digital camera Andor LUCA™.

### AFM imaging

Images of ejected DNA were obtained with an AFM (Asylum Research™, MFP-3D™) using a cantilever (AC160TS, Standard Si cantilever, Olympus) with a <10 nm tip. Fifty µl of a solution containing PBCV-1 particles or viral DNA (10 ng/µl), which was released from the capsids by osmotic shock, were incubated for 5 min on a smooth mica surface. The preparation was washed twice with 1 ml distilled water and dried by high air pressure (1 min, 2 bar). For imaging, the tapping mode was used which reduces sample exposure. The volume of particles was analysed using the particle analysing tool Image Processing Software, Image Metrology A/S with standard settings.

### Bioinformatic analysis of PBCV-1 genome

The experimental results indicated that one or more DNA binding proteins were involved in organizing the viral DNA in nearly equidistant units; if so, the mechanism would most likely require periodic binding motifs in the DNA. To identify protein-binding domains in PBCV-1 DNA we analyzed its genome sequence for periodic signals using our own code [43], [44], BioPyhton [45] and NumPy for Fast-Fourier-Transformations (FFT). First, we scanned the genome for 6 and 8 bp motifs including two wildcard characters at most, which match any nucleotide. We computed the Hamming distance of all possible fragments (330,742 fragments) to all possible, potential motifs (15,361 and 311,297 motifs, respectively). This produces a string of integers – representing the hamming distance - ranging from zero to *N*, where *N* is the motif length. Any periodic or semi-periodic structures in this data are revealed by Fourier analysis as peaks in the Fourier components. We restricted our analysis to small motifs because of computational constraints. Therefore, we could not detect signals for larger motifs directly, e.g., two binding motifs connected by a highly variable region. This prompted us to not look for one particular motif with the most pronounced peak in the Fourier spectrum. Instead, we counted how often a particular length scale appeared, averaged over all motifs. This protocol has an additional advantage because it also reveals periodicities of much larger motifs; e.g., consider the simple case of two motifs separated by some variable genomic region. Separately these two motifs would exhibit the same periodicity. If we analyze the data averaged over all motifs, however, we also uncover the “synchronization” of the motifs along the chain and therefore the correlated periodicity. We also analyzed the motifs that contributed most, for their relative content of nucleotide types.

### DNA bound proteins

In a further analysis we separated DNA bound proteins from the re-suspended pellet by SDS PAGE. Peptide map fingerprinting (PMF) of proteins from SDS-PAGE gel slices was performed using standard procedures, with treatment of the proteins with dithiothreitol and iodoacetamide followed by trypsin digestion. Peptides eluted from the gels were purified on ZipTip C18 columns (Millipore) and applied to a stainless steel target together with α-cyano-4-hydroxycinnamic acid as a matrix. The peptides were analyzed in a reflectron mode using a Shimadzu Biotech Axima Performance MALDI-TOF mass spectrometer. Calibration was via nearest neighbor external standards, using 8 peptides (Sigma Aldrich) with m/z from 757.4 to 3657.9. Mass lists from the individual PMF spectra were submitted to an in-house Mascot Server PMF search engine using the NCBInr database. The taxonomy was limited to *C. variabilis* and PBCV-1 virus. Additional search parameters were set to monoisotopic mass, charge 1+, maximum of 1 missed cleavage, peptide tolerance of 0.3 m/z and p<0.05. The root mean square (RMS) errors on the peptide mass matches ranged from 21–102 ppm. As a control, all searches were repeated using the decoy database generated by the Mascot Server software, using the same settings. In some cases, high energy CID MS/MS sequencing of the peptides was employed (using the same samples and instrumentation) to confirm protein identification.

### Virus purification scheme for the proteomic study

The virus was purified essentially as described previously [42] with the following modifications. Prior to sucrose density gradient separation, the virus-infected cell lysate was clarified by first incubating with 1% (v/v) NP-40 detergent at room temperature for 1–2 hr with constant agitation, concentrated by centrifugation in a Beckman Type19 rotor at 17,000 rpm, 50 min, 4°C. The pellet fraction was solubilized in virus storage buffer (VSB) (50 mM Tris-HCl pH 7.8), loaded onto a 10–40% (w/v) linear sucrose density gradient made up in VSB and centrifuged in a Beckman SW28 rotor for 20 min at 20,000 rpm at 4°C. The virus band was identified by light scattering, removed from the gradient and pelleted. The resuspended virus was “washed” with 50 µg/mL proteinase K in VSB for 4 hr at 25°C to disassociate and degrade contaminating proteins. The proteinase K treated virus was layered onto a 20–40% linear gradient of iodixanol (OptiPrep™, Axis-Shield, Oslo, Norway) in VSB and centrifuged 20,000 rpm in a Beckman SW28 rotor for 4 hr at 25°C for isopynic separation. The virus band was removed by side-puncture of the centrifugation tube, diluted approximately 10 fold with VSB, then concentrated by centrifugation in a Beckman Ti50.2 rotor at 27,000 rpm for 3 hr at 4°C. The pellet fraction was re-suspended in VSB, then filter sterilized with 0.45 µm cutoff membrane, and stored at 4°C.

### Whole virion proteome

Virion proteins were evaluated essentially as described [12]. Gradient purified, protease-washed PBCV-1 particles were disrupted with 1% SDS/5 mM dithiothreitol and the proteins were separated by SDS-PAGE. The gel was comprehensively evaluated for viral proteins by mass ion analyses of peptides eluted from trypsin-digested gel slices. MS/MS data were processed using Masslynx software to produce peak lists for database searches with MASCOT (Matrix Science). Database searches were done against the newly re-sequenced and annotated PBCV-1 genome (GenBank accession number JF411744.1). Protein identifications were based on random probability scores with a minimum value of 25, the value for p<0.05 confidence. Approximate, relative quantitation of the proteins was determined using the exponentially modified protein abundance index (emPAI) [40]. This method uses the number of observed peptides compared to the number of observable peptides giving a ratio that is directly proportional to relative abundance of the protein in the mixture when adjusted exponentially (emPAI = 10^Nobserved/Nobservable^−1). We assumed the major capsid protein (A430L) is present in 1440 copies per virion for these calculations and other protein abundances were estimated from this value.

## Supporting Information

Bioinformatics Analysis S1 **Application of Fast-Fourier-Transformation (FFT) protocol for detecting potential periodicities of binding motifs in the genome of dsDNA virus PBCV-1.**
(DOC)
