# Supplementary material for: Structural Organization of DNA in Chlorella Viruses
Source: PLoS One. 2012 Feb 16;7(2):e30133. doi: 10.1371/journal.pone.0030133 (PMC3281028; doi:10.1371/journal.pone.0030133)
Supplement: Bioinformatics Analysis S1 — Application of Fast-Fourier-Transformation (FFT) protocol for detecting potential periodicities of binding motifs in the genome of dsDNA virus PBCV-1. (DOC) [file pone.0030133.s001.doc]

**Bioinformatics Analysis**  **S1:**

General Theory

We want to analyze a genomic sequence written as a string of symbols g[n] for periodicities. Here n is an index ranging from 0 to N-1, counting the N nucleotides from the 5‘ to 3‘-end of the DNA. This string of characters will be transformed into numerical values according to different, but related mappings:

- Hamming distance-based. We compute for a given motif m of length M the hamming distance h[n] of g[n], here n runs from 0 to N-1-m. We allow for a wildcard character in the motif that matches any nucleotide.

- Exact match based. We set for a given motif m of length M the value of e[n] to 1 if the motif m matches the m characters starting at g[n] exactly, and 0 otherwise.

Any periodicity within the sequence can now be found in the spectrum of the Fourier series S[k] resulting from the original numerical sequence s[n] (here either s[n]=h[n] or s[n]= e[n]). The Fourier series expansion

is computed by the Fast-Fourier-Transform (FFT) method.

The spectrum is then obtained as T[k] := S[k] . S^*^[k]. If there exists any periodic signal within such a sequence g[n], then T[k] will have a peak at the particular „wave number“ k.

Significance of Signals

For h[n] we show a histogram of the largest Fourier spectrum entry for each of the motifs of length eight and six in figure 1. We had to rescale the values to account for the varying overall scales between motif lengths six and eight. Although the results vary in the left part between sequence lengths, the distinct peaks to the right (for > 0.8) shows that a significant periodic signal is present in some of the motifs and this property can be found for varying motif lengths.

**Figure 1:**

Histogram over all sequence motifs of length six (red) and eight (blue) with two wildcard characters at most. To compare directly we normalized the Fourier coefficients by the respective maximum. Clearly we see a distinct peak at higher values; indicating a significant signal in comparison to the background of „typical“, average motifs (normalized Fourier component < 0.8)

Periodicity in the genomic sequence

In figure 2 we show the main result for the test on the hamming distance transformation: a strong signal for periods of 9935bp and some minor peaks at other inverse frequencies.

| 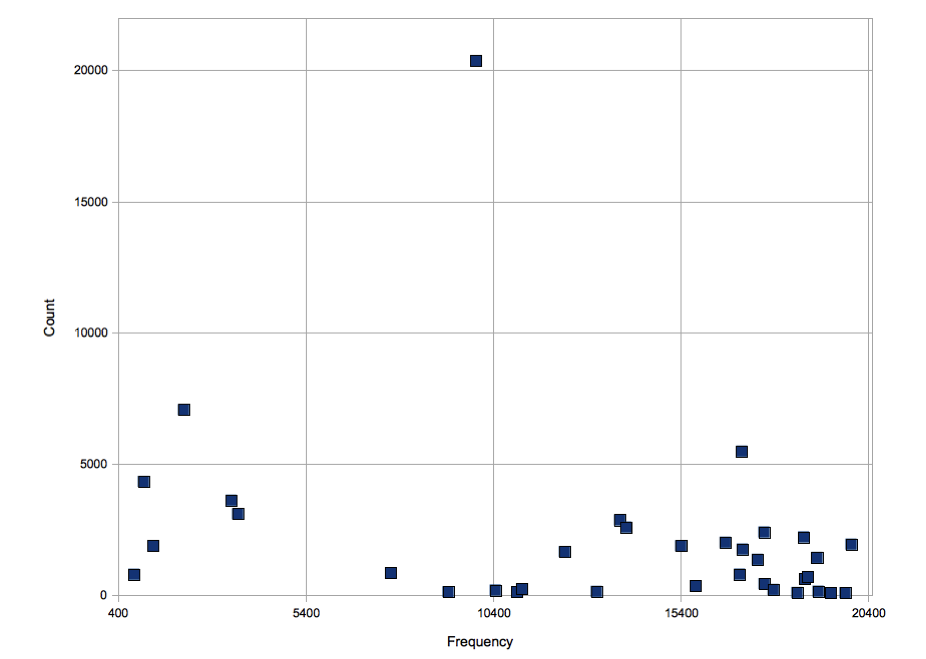 |  |
| --- | --- |

**Figure 2:**

Histogram of the leading inverse Fourier-frequency [in bp] for all sequence motifs of length eight (blue). The most pronounced peaks occur at 9935 bp, 2138 bp, and 17020 bp. Interpretation: a significant number of motifs show the exact same period of 9935 bp.

To verify the consistency of our method we proceeded to test whether the second, third, fourth,... largest component would give similar results. The results are shown in figure 3.

| 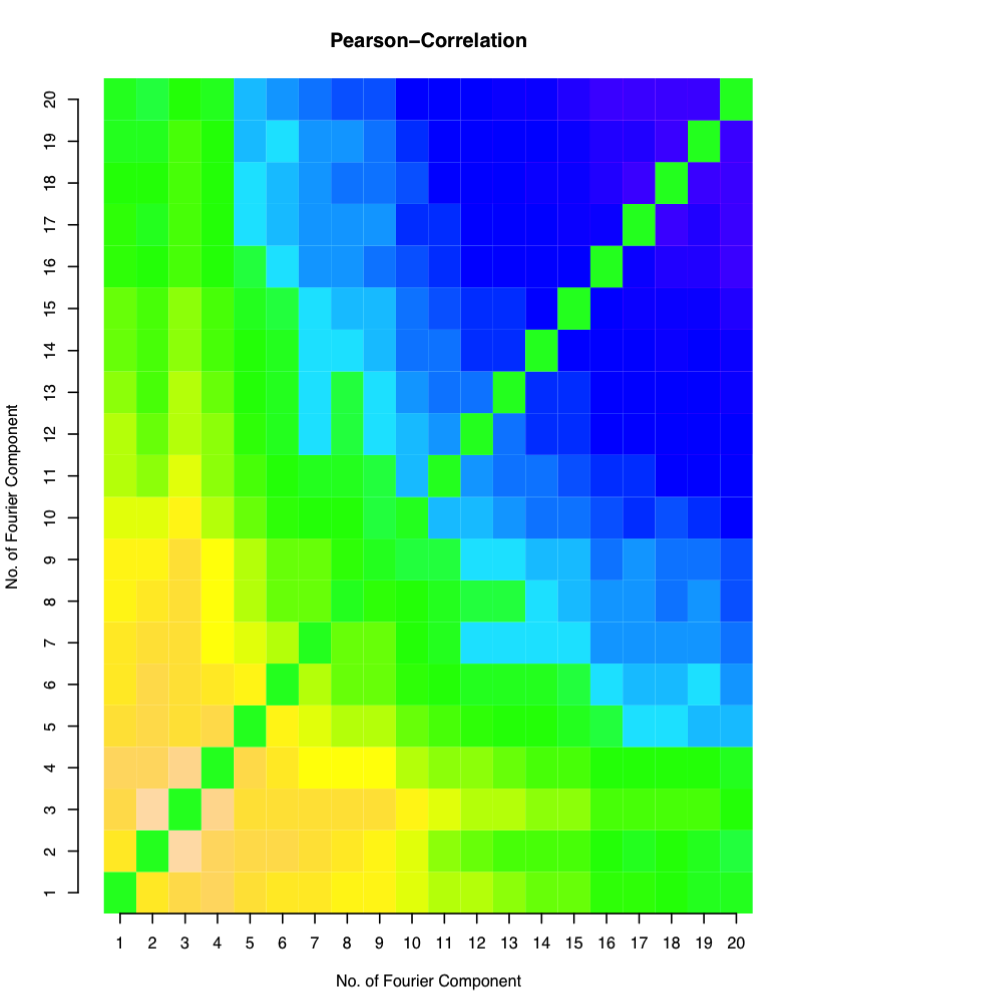 |  |
| --- | --- |

**Figure 3:**

The linear Pearson correlation coefficient between the Fourier spectrum heights between the first 20 leading Fourier components for all sequence motifs in the *h*[*n*] transformation. yellow=1.0, dark blue=0.5. Clearly the most important frequencies (no. 1,2,3...) give raise to the same classification and indicate the consistency of the methodology.

Results for exact matching

A first insight is obtained by looking at the distribution of the number of hits (for motif length six) within the chlorovirus PBCV-1 genome. The results in figure 4 provide proof that the genome is distinct from a random sequence, as the distribution is flat at the beginning - contrary to what one would expect for random sequences.

| 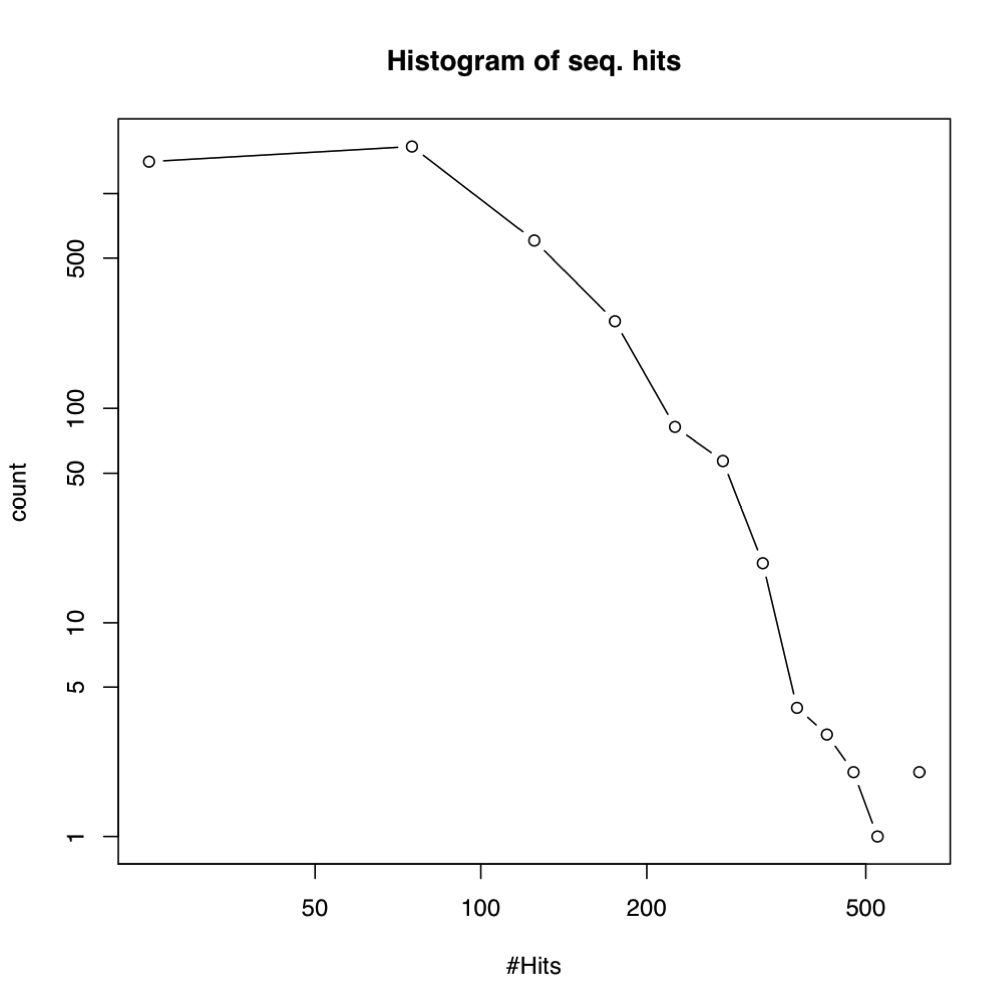 |  |
| --- | --- |

**Figure 4:**

Histogram of the number of times a length-6-motif was found *exactly* in the genome.

We note that the distribution of Fourier spectrum heights for e[n] is similarly pronounced to the one shown in figure 1 for the h[n]-transformation, thus large components indicate a sufficiently pronounced signal for periodicity. The most important frequencies are: 6835, 9571, 17300, 16801, 17489, 4409, 23423, 8831, 15648, 2064 [all values measured in bp].

The patterns which contributed significantly are as follows:

| TTGG** | CACAA* | CAACA* | CAAAC* | CAATC* | AC*ACT |
| --- | --- | --- | --- | --- | --- |
| TGTGG* | *TTGG* | CACA*A | CA*CAA | C*ACAA | *CACAA |
| GCACAA | GACCAA | C*AACA | *CAACA | GCAACA | CGAACA |
| GACACA | GTCAGA | CAAA*C | CAAT*C | ACAT*C | CA*AAC |
| C*AAAC | *CAAAC | GCAAAC | CGAAAC | CAA*TC | CA*ATC |
| AC*ATC | C*AATC | *CAATC | CAGATC | TGTG*G | TGT*GG |
| TTCTGG | T*GTGG | *TGTGG | TCTTGG | TTGCTG | TTCGTG |

For the h[n] case we found an interesting feature: all leading contributions result from patterns including all combinations of CG-occurrences. To show that this is a significant finding, we show in figure 5 the respective leading Fourier coefficient for all possible patterns (including two wild-cards).

| 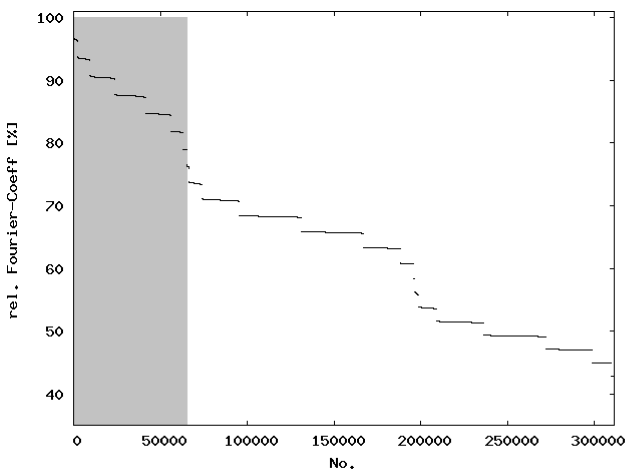 |  |
| --- | --- |

**Figure 5:**

The leading Fourier component for all patterns of length 8, including two wild-card characters. The gray shaded area represents the patterns without wild-cards. The first 256 patterns with contributions of some 100% can be identified with the 2^8^=256 possible combinations of CG-content.
